# The human embryo selection arena is associated with transposable element activity

**DOI:** 10.1371/journal.pbio.3002153

**Published:** 2023-06-20

**Authors:** Anna Osnato, Vincent Pasque, Laurent David

**Affiliations:** 1 KU Leuven-University of Leuven, Department of Development and Regeneration, Leuven Stem Cell Institute, Leuven, Belgium; 2 KU Leuven Institute for Single Cell Omics (LISCO), Leuven, Belgium; 3 Nantes Université, Inserm, CR2TI, Nantes, France; 4 Nantes Université, CHU Nantes, CNRS, Inserm, BioCore, Nantes, France

## Abstract

Our current understanding of early human development is limited. A study in *PLOS Biology* found a previously undefined group of cells that diverges from the main lineages and undergo apoptosis through the activity of young transposable elements.

Upon fertilisation, the human embryo undergoes several rounds of cleavage divisions and morphological changes generating the blastocyst, a cavitated structure that will implant in the uterus 7 days post fertilisation (dpf). Understanding how cell specification takes place in the human embryo has significant implications in improving human reproductive health, in particular in vitro fertilisation (IVF) success rate [1].

Before implantation, the zygote undergoes successive divisions and ultimately is composed of 3 lineages, namely, the trophectoderm (TE), epiblast (EPI), and primitive endoderm (PrE). Single-cell RNA sequencing (scRNA-seq) allowed the generation of expression atlases of early mammalian development, which was pivotal to identify molecular signatures associated with each fate. Nonetheless, the datasets generated are large and the analysis pipelines are constantly improving, giving opportunities for refined analysis of the scRNA-seq datasets. A particular challenge lies in identifying cells that are not clearly aligning with one fate, often dubbed “intermediate”. Careful in-depth analysis of those “intermediate” cells is key to account for distinguishing biological features from technical and biological noise.

Here, Singh and colleagues reanalyse multiple independent scRNA-seq datasets [2–4] of early human development focusing specifically on a cell population that is not clearly linked to a fate, naming this population “REject cells” (REjects), since those noncharacterised cells express high levels of Retro Elements. REjects segregate after embryonic gene activation, represent as much as 20% of the early embryo, and undergo apoptosis shortly after inner cell mass (ICM) specification (end of 5 dpf). Dimension reduction methodologies and lineage decision trajectory show that these cells do not follow the developmental path of neither ICM nor TE through the progression of blastocyst formation and therefore do not express lineage-specific markers, suggesting that they are likely excluded from the lineage specification process. Programmed cell death appears to be involved, as REjects express high levels of TP53-dependent/damage-response markers and preapoptotic signals, clustering apart from ICM or TE progenitors, confirming that the REjects are not engaged in neither of those lineages (Fig 1).

**Fig 1:**
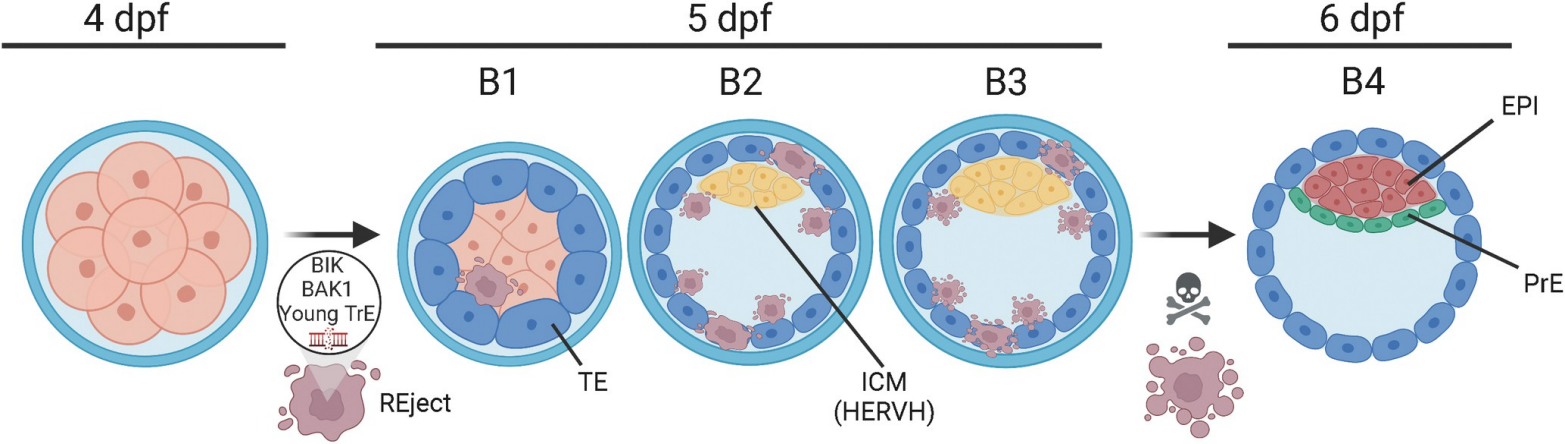
Proposed timing of selection for REject cells during human embryo development. REjects segregate at the blastocyst stage and undergo apoptosis at the end of 5 dpf/E5. They express the apoptosis-inducing factor BIK, genes associated with programmed cell death such as BAK1, and their selection is regulated by young transposable elements activity. The ICM is instead characterised by the activity of an old nontransposing endogenous retrovirus (HERVH) that acts to suppress young transposable elements. B1, B2, B3, blastocyst stages; BIK, BCL2-interacting killer; dpf, days post fertilisation; EPI, epiblast; HERVH, human-specific endogenous retrovirus H; ICM, inner cell mass; PrE, primitive endoderm; TE, trophectoderm; TrE, transposable elements. Figure generated using BioRender.com.

Of great importance, identification of the REjects permitted clear identification of ICM cells. Moreover, the authors reveal a previously unknown role for transposable elements. REjects are associated with the activity of young transposable elements, which include potentially mutagenic transposons. In contrast, the developing ICM only expresses old (no longer able to transpose) human-specific endogenous retrovirus H (HERVH), which is capable of suppressing active transposable elements by modulating the expression of retroviral inhibitors. Among these transposable elements restricting factors, APOBEC3s seem to be indeed uniquely expressed in the ICM and proposed to suppress young transposable elements activity.

The work of Singh and colleagues sheds new light on early human development [5]. The study provides evidence supporting sequential lineage specification in the human embryo, where the first cell fate decision segregates TE from ICM, and the second cell fate decision within ICM cells specifies the EPI and PrE [6,7]. In identifying REjects, a previously unannotated cluster, the authors highlight the importance of taking undefined cell types into account within high-dimensional datasets. Indeed, attributing an apoptotic signature to REjects enables their removal from subsequent analyses, reducing the noise in scRNA-seq preimplantation datasets and allowing to define a more robust set of markers that characterises the human ICM, devoid of DNA damage hallmarks. Interestingly, using a new mathematical framework, such stringent ICM markers have been also reported in a recent publication [8] and seem to be conserved in nonhuman primates. The identification of ICM markers is of great importance, as a deeper understanding of the regulation of lineage markers during preimplantation development is key to improve embryo cultures in vitro and consequently IVF success rates.

Singh and colleagues also suggest that endogenous retroviruses such as HERVH represent a key defence mechanism for the developing embryo against potential DNA damage, linking transposable elements to their known role in pluripotency [9]. It is known that chromosomal instability and mosaicism are present in early human embryos, and in some cases, aneuploid cells may be selected out, suggesting that cell competition takes place [10]. In this study, Singh and colleagues propose a “selection arena” model, in which a set of cells with distinct markers die while the others specifically survive, through transposable elements activity. It is yet to be defined whether the activity of these transposable elements is the cause of this selection (e.g., REjects die because the activity of these transposable elements induces damage), or rather it is just the way apoptosis is induced (e.g., aneuploid cells are committed to die through up-regulation of mutagenic/young transposable elements).

Finally, REjects seem to be absent in mice at similar developmental time frames, highlighting interspecies differences that need to be taken in account when translating animal model findings to humans, and emphasising the importance of developing relevant models to study human early development. On this note, it is possible that REjects apoptosis could be an artefact from in vitro culturing of human embryos. However, their reproducibility in different datasets and predicted presence in nonhuman primates would dismiss this hypothesis. To strengthen these findings, it will be important to identify more precisely how HERVH suppresses young transposable elements activity in vivo and the mechanisms that regulate induced apoptosis. It will be also interesting to investigate whether transposable elements activity is a developmentally conserved mechanism of gene regulation, as it could perhaps also apply to cell fate decisions. In other words, in parallel to selecting out REjects, repressing transposable elements might be necessary for further lineage specification, as it has been proposed that ICM markers such as IFI16 could inhibit the burst of transposable elements expressed at the morula stage [7,11].

Overall, the work by Singh and colleagues in this issue of *PLOS Biology* highlights the importance of carefully mining and correctly annotating large omics datasets, and to thoughtfully consider “intermediate” cells to define whether they are representing transitions or aberrant cell fates or states that would undergo apoptosis. Through the identification of REjects, they describe an apoptosis-mediated mechanism for cell selection in the early human embryo correlated with transposable elements activity. This shows that abnormal fates, potentially related to aneuploidies, are extremely frequent in all embryos and hints at the way that aneuploid cells may be selected out.

## Abbreviations

dpf: days post fertilisation
EPI: epiblast
HERVH: human-specific endogenous retrovirus H
ICM: inner cell mass
IVF: in vitro fertilisation
PrE: primitive endoderm
scRNA-seq: single-cell RNA sequencing
TE: trophectoderm
